# Plasma pharmacokinetics of the combination of potassium chloride extended-release tablets and potassium citrate granules in patients with cardiovascular emergencies

**DOI:** 10.3389/fcvm.2025.1636090

**Published:** 2026-01-06

**Authors:** Xinmei Li, Minghong Qu, Haizhi Li, Te Li

**Affiliations:** Department of Pharmacy, Fuwai Yunnan Hospital, Chinese Academy of Medical Sciences/Affiliated Cardiovascular Hospital of Kunming Medical University, Kunming, China

**Keywords:** cardiovascular disease, emergency patient, non-compartmental model, plasma potassium parameters, potassium

## Abstract

**Objective:**

To simulate plasma pharmacokinetics (PK) parameters of combining potassium citrate granules and potassium chloride extended-release tablets using real-world data, guiding optimal dosing and timing for cardiovascular emergency patients.

**Methods:**

A retrospective study analyzed PK characteristics in patients receiving both drugs at Fuwai Yunnan Hospital from January 1 to March 31, 2024, using non-compartmental analysis (NCA).

**Results:**

Among 330 patients, the *T*_max_ values for 1-dose, 2-dose, and 3-dose administrations were 4.65 h, 6.00 h, and 6.11 h. The half-life (*t*_1/2_) was 1.33 h, 13.53 h, and 6.11 h, respectively. Each dose elevated plasma potassium by 0.31 ± 0.05 mmol/L. The limited areas under the plasma curve (AUC_limit_) were 30.37 mmol-h/L, 106.89 mmol-h/L, 141.74 mmol-h/L. Co-administered dose and AUC_limit_ were linearly related (R^2^ = 0.9554).

**Conclusion:**

PK data fit a 1-compartment model, supporting safe, rational potassium supplementation in cardiovascular emergencies.

## 1 Introduction

Potassium dysregulation or abnormal potassium metabolism is common in patients with cardiovascular disease in the emergency setting due to the widespread use of diuretics (1). Persistent abnormalities in plasma potassium may lead to severe or life-threatening arrhythmias and death. It has been shown that the ideal plasma potassium level for patients with cardiovascular disease is 4.0–5.0 mmol/L (health: 3.5–5.0 mmol/L), with an optimal potassium value of 4.2 mmol/L (2). In clinical practice, a dual-strategy approach is sometimes adopted for rapid and sustained potassium replenishment. This involves co-administering a fast-absorbing preparation (e.g., potassium citrate granules)[1.46 g, Weight Equivalent (WEq):0.5589 g]) to promptly address the potassium deficit, alongside a slow-absorbing extended-release preparation (e.g., potassium chloride extended-release tablets)(1.0 g, WEq:0.5245 g) to maintain stable plasma levels and minimize gastrointestinal irritation (3). However, direct evidence supporting the pharmacokinetic (PK) profile, safety, and dosing rationale of this specific combination regimen in cardiovascular emergency patients is lacking. Previous PK studies have primarily focused on single formulations in healthy volunteers or used urinary excretion endpoints per FDA guidance for bioequivalence assessment (4, 5). Therefore, this study aims to characterize the plasma PK of this combined regimen in a real-world cohort of cardiovascular emergency patients, providing evidence to guide its rational use.

The pharmacokinetics of potassium in humans is Unusual (3). Plasma potassium levels are regulated by homeostasis *in vivo* and their absorption was hard assessed by conventional plasma pharmacokinetics (PK), the US FDA recommends the use of urinary PK parameters (e.g., maximal excretion rate, Rmax, and cumulative excretion over 24 h, Ae0-24 h) as the criteria for bioequivalence (BE) evaluation. Only three studies of plasma potassium have been reported. (1) In healthy people, after oral administration of 64 mEq of potassium chloride solution, the time to peak (*T*_max_) was 1.0 h and the increase in peak concentration (*C*_max_) was 1.7 meq/L (4). (2) the *T*_max_ was 1.0 h and the increase in *C*_max_ was 0.8 meq/L after potassium supplementation of 50 mEq (5). (3) In intensive care unit (ICU) patients, plasma potassium could be increased by 0.245 mmol/L by per 20 mmol administered via the intravenous and enteral routes (6). For clinical practice, the plasma potassium concentration is more responsive to whether potassium supplementation achieves the target effect than the excretion in the urine. Fluctuations in plasma potassium have not been reported in disease states, especially in patients in cardiovascular emergencies. Non-atrial model (NCA) is a conventional method to analyze drug pharmacokinetics, providing key parameters such as *T*_max_ and *C*_max_. In order to further explore the rationality and safety of potassium supplementation with multi-group combination drugs, this study attempted to use NCA to analyze the plasma potassium pharmacokinetic parameters of patients with cardiovascular emergencies.

## 2 Manuscript formatting

### 2.1 Materials and methods

#### 2.1.1 Patients' characteristics

The retrospective study analyzed PK characteristics in patients receiving both drugs. From January 1, 2024, to March 31, 2024, the patients who were prescribed both potassium chloride sustained-release tablets and potassium citrate granules were enrolled in Fuwai Yunnan Hospital, Kunming, China from Hospital Information System. The inclusion criteria, according to the enrollment criteria: (1) adult patients (>18 years old); (2) emergency patients with cardiovascular diseases (e.g., angina, arrhythmia, and heart failure); (3) patients who were co-administered potassium chloride sustained-release tablets and potassium citrate granules. The exclusion criteria were patients: (1) on diuretics in the emergency setting; (2) with communication problems or other problems such as visual or hearing impairment. The potassium citrate granules was purchased from Changchun Beihua Pharmaceutical Co., Ltd. (Changchun, Jilin, China)(0.5 g, Approval Number: H22024249). The potassium chloride extended-release tablets was purchased from Shanghai haihong Group Chaohu C-Dragon Pharmaceutical Co., Ltd. (Shanghai, China)(2 g, Approval Number: H34021120). Physicians prescribed three dosing regimens: 1-dose (potassium chloride extended-release tablets 0.5245 g + potassium citrate granules 0.5589 g), 2-dose (1.049 g + 1.1178 g), and 3-dose (1.5735 g + 1.6767 g). Each dose was administered at 0.5-hour intervals, with a maximum of three doses. The study protocol was approved by the Ethics Committee of Fuwai Yunnan Cardiovascular Hospital, Kunming, China (No.2024-046-01). The human samples used in this study were acquired from a by- product of routine care.

#### 2.1.2 Statistical analysis

Plasma potassium parameters were calculated using the WinNonlin 7.0 Software (Pharsight Corporation, Mountain View, CA, USA) via non-compartmental modeling. Linear regression methods were employed to analyze the relationship between the limited areas under the plasma curve (AUC_limit_) and co-administered dose. The experimental data were expressed as mean ± standard deviation. Statistical analyses were using the GraphPad Prism 6 software (San Diego, CA, USA).

### 2.2 Result

#### 2.2.1 Patients' characteristics

According to the criteria, 330 emergency patients who were prescribed both potassium chloride sustained-release tablets and potassium citrate granules were enrolled. The characteristics of the 330 patients are presented in Table 1. Due to subtle physiological adjustments in muscle mass, hormones, and kidney function, women's serum potassium levels are on average approximately 0.1–0.2 mmol/L lower than men's. Table 1 presents gender-specific subgroup data, though no statistically significant differences were observed in pre-co-administered values. Post-co-administered values is statistically significant differences(P < 0.05).

**Table 1:** Clinical characteristics of 330 emergency patients before and after co-administered potassium chloride sustained-release tablets and potassium citrate granules.

Characteristic		Male group (*n* = 182)	Female group (*n* = 148)
Age, year		56.72 ± 21.80	60.36 ± 15.46
Pre-co-administered values	plasma potassium concentration, mmol/L	3.57 ± 0.31	3.55 ± 0.25
Post-co-administered values	plasma potassium concentration, mmol/L	3.95 ± 0.42	4.20 ± 0.45
potassium chloride sustained-release tablets and potassium citrate granules	1-dose administration group	54 (29.67%)	38 (25.68%)
	2-dose administration group	92 (50.55%)	37 (25%)
	3-dose administration group	36 (19.78%)	73 (49%)

**Figure 1:**
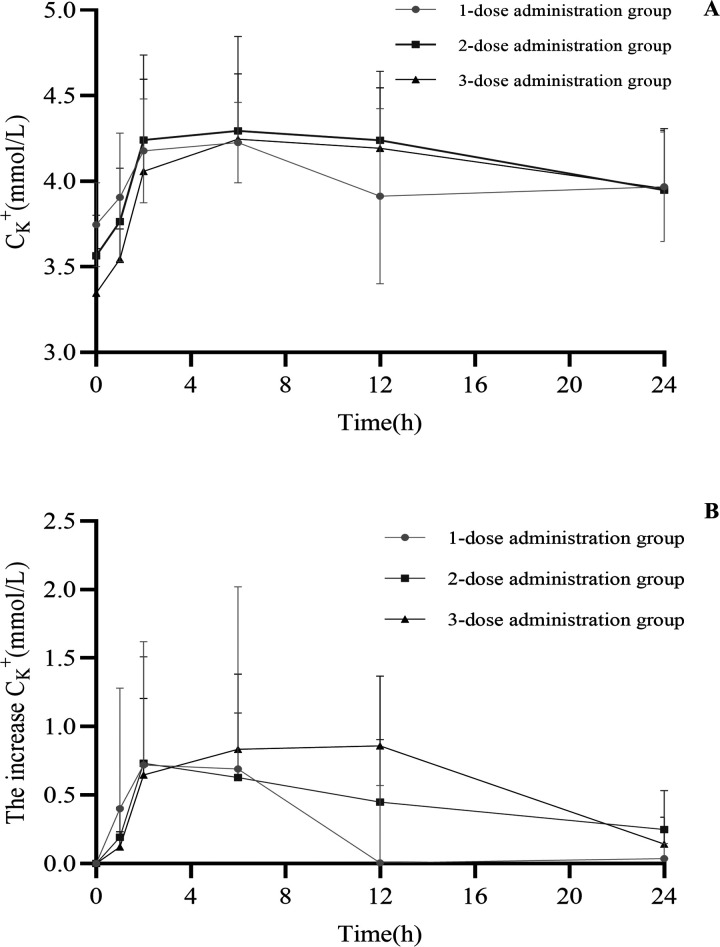
Mean concentration–time curves of the plasma potassium concentration **(A)** and the increase in plasma potassium concentration **(B)** in the emergency patients after co-administration.

Plasma potassium was elevated in emergency patients using co-administration potassium supplements (potassium chloride extended-release tablets and potassium citrate granules). The mean concentration–time curves of potassium concentration in the plasma of emergency patients were determined, as shown in Figure 1A. The patient's plasma potassium concentration before potassium supplementation (1-dose administration group, 2-dose administration group, and 3-dose administration group) was 3.70 ± 0.24 mmol/L, 3.54 ± 0.29 mmol/L, and 3.36 ± 0.26 mmol/L. The 12-hour time point showed the largest difference in the increase in plasma potassium concentration, which is 0.005 ± 0.56 mmol/L, 0.45 ± 0.46 mmol/L, and 0.86 ± 0.51 mmol/L, respectively (Figure 1B) (*P* < 0.0001).

#### 2.2.2 Non-compartmental model

The pharmacokinetic parameters for plasma potassium concentrations are presented in Table 2. The following parameters were calculated by WinNonlin Software. Each dose elevated plasma potassium by 0.31 ± 0.05 mmol/L. The AUC_limit_ were: 30.37 mmol·h/L, 106.89 mmol·h/L, and 141.74 mmol·h/L. Linear regression of co-administered dose and AUC were showed as follows:

**Table 2:** PK parameters of the plasma potassium concentration in the emergency patients after co-administration (mean ± SD).

Group	*C*_0_ (mmol/L)	*T*_max_ (h)	*C*_max_ (mmol/L)	*ΔC* (mmol/L)	*K*_a_ (per hour)	*t*_1/2_ (h)	AUC_limit_ (mmol·h/L)
1-dose administration group (extended-release tablets 0.5245 g and granules 0.5589 g)	3.75 ± 0.24	4.65	4.11 ± 0.35	0.36	0.36	1.33	30.37
2-dose administration group (extended-release tablets 1.049 g and granules 1.1178 g)	3.56 ± 0.29	6.00	4.14 ± 0.49	0.58	0.37	13.53	106.89
3-dose administration group (extended-release tablets 1.5735 g and granules 1.6767 g)	3.34 ± 0.26	6.11	4.15 ± 0.55	0.81	0.32	6.55	141.74
Literature 1 (granules 64 mEq)	4.6 ± 0.2	1.0	5.8 ± 0.5	1.7	-	-	-
Literature 2 (granules 50 mEq)	4.1 ± 0.1	1.0	4.9 ± 0.2	0.8	-	-	-

*K*_a_, absorption rate constant; *t*_1/2,_ half-life.

AUC=55.69 × times of dose-18.37 (R^2^ = 0.9554).

where times of dose including 1, 2, or 3 represent 1-dose administration group(8.7583 mol), 2-dose administration group(17.5166 mol), or 3-dose administration group(26.2749 mol).

### 2.3 Discussion

#### 2.3.1 The need for dietary potassium in patients with cardiovascular disease

Due to the widespread use of diuretics, hypokalemia often occurs in acute patients with cardiovascular disease. We suggest that patients with cardiovascular disease who use diuretics, especially potassium-excreting diuretics, could use plus supplements on a daily basis. Studies have shown that a low-potassium diet activates the potassium-sensing signaling pathway in the renal distal convoluted tubule, increasing the activity of the sodium-chloride cotransporter and sodium retention, thereby elevating blood pressure and enhancing salt sensitivity (7, 8). In contrast, increasing potassium intake could lower blood pressure by enhancing AngII-stimulated aldosterone secretion (without affecting cardiovascular responses), primarily through a diuretic mechanism (9). Hypertensive patients exhibit greater sensitivity to increased potassium intake compared to normotensive individuals, with particularly pronounced benefits for those on a high-salt diet (10). A high-potassium diet effectively reduces blood pressure in individuals with prehypertension and hypertensionand reverses endothelial dysfunction induced by high sodium intake, mitigating vascular damage (11). Additionally, dietary potassium not only improves blood pressure (12, 13). but also enhances glucose tolerance and insulin resistance (14). These studies confirm the multifaceted cardiovascular and metabolic benefits of dietary potassium. This might reduce the risk of developing serious cardiovascular disease.

#### 2.3.2 Plasma PK of co-administration in emegency patients

The conventional opinion is that fluctuations in plasma potassium caused by oral potassium-supplementing preparations are difficult to detect, and that intravenous administration without an absorptive phase is easier for raising plasma potassium. Oral extended-release preparations could delay potassium release in order to minimize gastrointestinal irritation (3). In this study, we found a linear relationship between the administered dose and plasma AUC in patients with cardiovascular emergencies. This provides an evidence-based basis for potassium supplementation in these patients. To rapidly replenish a patient's plasma potassium, physicians use the strategy of fast-absorbing granules preparations (potassium citrate granules) and slow-absorbing extended-release preparations. This combination achieves rapid, prolonged potassium supplementation.

The *T*_max_ of the combination was prolonged to 4–6 h compared to the 1 h *T*_max_ of granules KCl in the literature (4, 5). Patient's plasma potassium rose rapidly over a 2 h period and *K*_a_ averaged 0.35. Drug absorption was at 2–6 h for 3-dose administration due to the extended-release dosage form. 3-dose administration approximated an equilibrium of absorption and elimination at 6–12 h, with Ka = Ke = 0.10. It can be hypothesized that the Ka of KCl extended-release tablets alone might be 0.10 with a t1/2 of 6.93 h. Based on this, if the goal is to maintain target plasma potassium levels, the recommended dosing interval should approximate the apparent half-life of the extended-release component multiplied by the number of doses administered. The number of administrations can be calculated based on the patient's pre-administration plasma potassium level. Therefore, (1) 1-dose administration: the need to maintain attained plasma potassium concentrations suggests that a second dose be given after 6.93 h. (2) 2-dose administration: it is recommended to be re-dosed after 13.86 h to maintain the standardized. (3) 3-dose administration: it is recommended to be re-dosed after 20.79 h to maintain the standardized. Further studies would be needed to confirm the *K*_a_ results for KCl extended-release tablets.

Among our 330 patients, 8 cases (2.4%) developed transient serum potassium elevation >5.0 mmol/L after administration, with the highest value being 5.3 mmol/L. All were asymptomatic, received no specific treatment, and returned to normal levels during subsequent monitoring. This relatively low incidence of hyperkalemia may be attributed to the following factors: (1) The extended-release formulation avoided rapid spikes in serum potassium; (2) The combination of liquid and solid formulations provided a relatively stable serum potassium profile; (3) Close monitoring of emergency patients.

There are some limitations of this study. Patients using diuretics were not selected during the study, reducing the interference of the elimination phase. Further studies are needed to investigate the effect of diuretics on potassium-supplementing medications in real-world.

## Data Availability

The raw data supporting the conclusions of this article will be made available by the authors, without undue reservation.
